# Dataset of cognitive behavioral intervention for persons living with HIV in China: A randomized pilot trial

**DOI:** 10.1016/j.dib.2020.105459

**Published:** 2020-03-31

**Authors:** Shuyu Han, Yan Hu, Zheng Zhu, Bei Wu

**Affiliations:** a School of Nursing, Fudan University, Shanghai 200032, China; b Rory Meyers College of Nursing, New York University, New York, USA

**Keywords:** HIV, AIDS, Cognitive behavioural therapy, Depression, Anxiety, Medication adherence, Randomized controlled trial

## Abstract

Globally, persons living with HIV (PLWH) are vulnerable to depressive and anxious symptoms [1]. Cognitive behavioural therapy (CBT) is one of the first-line mental health treatment strategies for PLWH [2–3]. However, structured and systematic cognitive behavioural intervention (CBI) is rare for PLWH in China. This data article presents the raw data of a parallel two-arm randomized controlled trial investigating the preliminary effects of CBI on depression, anxiety, medication adherence, quality of life, and CD4 lymphocyte counts for PLWH in China. Twenty PLWH who aged ≥18, were undergoing antiretroviral therapy (ART), and scored the Patient Health Questionnaire-4 (PHQ-4) ≥2 were recruited face-to-face and randomly assigned to groups based on computerized random number generation. Intervention participants received a tailored group-based 10-week-long CBI. Control participants only took laboratory tests and received free ART medication. The data includes demographic variables, exposure variables and outcomes. The outcomes were repeated-measured at baseline (T0), after the intervention (T1), and after 6 months of follow-up (T2). We assessed depression and anxiety via the Hospital Anxiety and Depression Scale (HADS), quality of life via the WHOQOL-HIV BREF, medication adherence via self-report adherence, the visual analog scale (VAS) and the medication possession ratio (MPR). CD4 lymphocyte counts were available on participants’ medical records. The main manuscript of this dataset is “cognitive behavioral intervention for persons living with HIV in China: a randomized pilot trial” (Han et al., submitted for publication) [4].

Specifications table

**Table:** 

Subject	Nursing and Health Professions
Specific subject area	Mental Health Nursing, AIDS Care
Type of data	Tables
How data were acquired	Questionnaires include a standardized demographic questionnaire, a 4-item Patient Health Questionnaire-4 (PHQ-4) for screening participants’ depression and anxiety, a 14-item Hospital Anxiety and Depression Scale (HADS), a 31-item WHOQOL-HIV BREF, a 2-item self-report adherence questionnaire, and numerical 0–100 visual analogue scale (VAS). We asked two questions for self-reported medication adherence. ①In the past month, did you miss taking medication or mistakenly take medication? ②In the past month, did you delay or take medication ahead of time by more than 2 h? If a participant answered “no” to both questions, we defined him/her as having good adherence; otherwise, the participant belonged to modest adherence category. The questionnaire is provided as a supplementary file.
	Every participant was receiving free antiretroviral therapy (ART) under the Chinese National Policies of “Four Frees and One Care for HIV/AIDS” [1], [2], [3], [4], [5]. They received routine follow-up every 3 months in the Shanghai Public Health Center Affiliated with Fudan University outpatient center, which included taking routine blood tests, urine tests, and receiving free ART medication. Their visit records and laboratory test results could be acquired from their medical records. After completing all the follow-ups, we checked every participant's latest four visit records to calculated their medication possession ratio (MPR) at T0, T1 and T2. The CD4 lymphocyte count was also acquired from medical records. Approved by the Research Ethical Committee both in School of Nursing, Fudan University and Shanghai Public Health Center Affiliated with Fudan University, we were allowed to login the in the archives system and check medical records.
Data format	Raw data
Parameters for data collection	The parameters included depression, anxiety, quality of life, medication adherence, CD4 lymphocyte count.
Description of data collection	We invited two nurses in the outpatient department to help us collect data. Neither of them participated in the intervention, so the data collection was blinded. The data collectors distributed paper questionnaires or let participants scan a QR code to fill out the questionnaire online. Data collectors checked the paper questionnaire on the spot and gave the questionnaire back if the patients had missed any questions. Online questionnaires could be submitted only after answering all the questions. Medical records were checked on the computers in the hospital to obtain patients’ follow-up intervals and CD4 lymphocyte counts.
Data source location	Shanghai Public Health Center Affiliated with Fudan University, Shanghai, China
Data accessibility	All the data for this randomized controlled trial are accessible in this data article.

## Value of the data

• The data can provide better knowledge for researchers and policymakers to evaluate the prospects of CBT for PLWH care in China.
• The data can provide reference for future research study design in the field of PLWH mental health care in China.
• The data can provide evidence for nursing practice of providing CBI for PLWH in China.
• The data may influence the model of mental health services for PLWH in China in the long term.

## 1 Data description

Tables 1–6 contain all the raw data of the randomized controlled trial. Baseline demographic information is shown in Tables 1 and 2. Tables 3–5 present outcomes data at T0, T1 and T2 respectively. Table 6 presents the attendance rates of the intervention group.

**Table 1:** Demographic variables of the study.

ID	Age	Gender	Household register	Race	Education	Religion	Employment status	Marital status
1	44	1	1	1	6	2	1	2
2	26	1	2	1	4	1	1	2
3	36	1	1	1	5	2	1	2
4	48	1	2	1	5	1	4	1
5	35	1	2	1	6	2	1	2
6	32	1	1	1	5	1	1	2
7	42	1	2	2	6	2	1	2
8	43	1	2	1	2	1	1	2
9	55	1	1	1	6	1	1	1
10	29	1	2	1	5	2	1	1
11	27	1	1	1	5	1	1	2
12	38	1	1	1	5	1	1	2
13	30	1	1	1	6	1	1	2
14	35	1	2	1	5	1	1	2
15	37	1	2	1	5	2	1	2
16	37	1	2	1	4	1	1	1
17	24	1	2	1	5	2	1	2
18	25	1	2	1	5	2	2	2
19	35	1	2	1	5	1	1	2
20	45	1	1	1	4	2	1	2

Note: Patients 1–10:intervention group; Patients 11–20:control group.

Gender: 1 = Male; 2 = Female.

Household register: 1 = Shanghai; 2 = Non-Shanghai.

Race: 1 = Han; 2 = Minority.

Education: 1 = Primary school or less; 2 = Junior high school; 3 = Senior high school; 4 = College; 5 = University; 6 = Master or higher.

Religion: 1 = No; 2 = Yes.

Employment status: 1 = Employed; 2 = Unemployed; 3 = Sick leave; 4 = Retired.

Marital status: 1 = Married; 2 = Single.

**Table 2:** PHQ-4 and HIV-related variables.

ID	PHQ-4	Transmission mode	CD4 lymphocyte count(/µL)	HIV-RNA	Years of HIV diagnosis	Years of receiving ART
1	4	1	376	0	1	1
2	3	1	370	0	1	1
3	6	1	266	0	1	1
4	4	2	197	0	2	2
5	5	1	253	0	6	6
6	5	1	356	0	6	6
7	10	1	768	0	8	7
8	4	1	443	0	8	7
9	5	1	409	0	9	8
10	6	3	372	0	4	4
11	2	3	433	0	1	1
12	4	1	509	0	7	2
13	4	1	339	0	2	2
14	5	1	549	0	3	3
15	2	1	295	0	3	3
16	10	1	436	0	4	4
17	3	1	539	0	4	4
18	5	1	481	0	4	4
19	9	1	265	0	5	5
20	4	4	449	0	5	5

Note: Patients 1–10: intervention group; Patients 11–20: control group.

Transmission mode: 1 = Homosexual behavior; 2 = Heterosexual behavior; 3 = Unknown; 4 = Other.

HIV-RNA: 0 = Undetectable; 1 = Detectable.

Years of HIV diagnosis: how many years has the participants been diagnosed with HIV infection?

Years of receiving ART: how many years has the participants been taking ART?

**Table 3:** Outcomes at baseline (T0).

ID	Anxiety	Depression	Negative emotion	Physical domain	Psychological domain	Independence domain	Social domain	Environment domain	Spirituality domain	Quality of life	VAS	Self-report adherence	MPR	MPR (binary)	CD4(/µL)
1	13	10	23	18	21	19	15	38	17	136	100	2	1.03	2	376
2	11	11	22	16	19	15	17	30	14	119	100	2	1.06	2	370
3	17	20	37	11	8	12	12	21	13	83	100	2	0.99	2	266
4	15	11	26	17	21	16	18	36	20	137	90	2	0.99	2	197
5	14	10	24	11	17	11	14	28	18	104	95	1	1.22	2	253
6	13	15	28	14	14	12	13	23	16	97	98	1	1.01	2	356
7	22	14	36	12	16	17	14	21	12	98	95	1	1.17	2	768
8	15	15	30	14	14	14	12	28	14	102	85	1	1.13	2	443
9	17	12	29	16	19	16	14	31	14	118	100	1	1.01	2	409
10	13	11	24	14	15	15	14	26	15	105	98	1	1.00	2	372
11	10	13	23	18	17	16	17	34	18	128	100	2	1.00	2	433
12	14	11	25	14	16	15	15	34	17	119	100	2	0.99	2	509
13	15	11	26	15	19	17	18	30	16	124	90	1	0.91	1	339
14	15	10	25	17	18	13	12	31	17	116	100	2	1.38	2	549
15	13	10	23	15	17	14	16	31	18	118	95	2	1.14	2	295
16	23	16	39	12	12	11	9	16	12	78	100	2	0.92	1	436
17	14	11	25	15	15	12	11	26	19	105	98	1	1.17	2	539
18	13	16	29	13	15	14	12	29	17	105	80	1	1.00	2	481
19	17	14	31	13	14	11	13	25	13	95	95	1	0.97	2	265
20	13	11	24	20	16	14	13	31	20	123	95	1	0.92	1	449

Note: Patients 1–10: intervention group; Patients 11–20: control group.

Anxiety: range from 7 to 28.

Depression: range from 7 to 28.

Negative emotion = Anxiety+Depression, range from 14 to 56.

Physical domain: range from 4 to 20.

Psychological domain: range 5 to 25.

Independence domain: range from 4 to 20.

Social domain: range from 4 to 20.

Environment domain: range from 8 to 40.

Spirituality domain: range from 4 to 20.

Quality of life = Physical domain+Psychological domain+Independence domain+Social domain+Environment domain+Spirituality domain+2 general items, range from 31 to 155.

VAS: visual analogue scale, range from 0 to 100.

Self-report adherence:1 = modest,2 = good.

MPR: medication possession ratio = time interval between two prescriptions/90.

MPR(binary):1 = modest(MPR<0.95),2 = good(MPR≥0.95).

**Table 4:** Outcomes after intervention (T1).

ID	Anxiety	Depression	Negative emotion	Physical domain	Psychological domain	Independence domain	Social domain	Environment domain	Spirituality domain	Quality of life	VAS	Self-report adherence	MPR	MPR (binary)	CD4 (/µL)
1	11	8	19	18	21	19	15	35	17	133	100	2	1.1	2	665
2	12	14	26	17	18	16	13	27	10	107	100	2	0.94	1	340
3	17	13	30	12	13	12	12	22	13	89	100	2	0.99	2	318
4	7	9	16	20	24	14	16	39	18	140	100	2	0.99	2	488
5	7	8	15	18	21	16	16	32	20	131	100	2	0.81	1	488
6	13	14	27	14	15	14	13	26	14	102	90	2	1.03	2	386
7	14	12	26	17	22	19	14	29	15	123	99	2	0.99	2	690
8	18	15	33	12	15	14	12	28	11	97	95	2	1.27	2	333
9	14	14	28	16	19	17	14	33	16	123	90	2	0.99	2	453
10	13	10	23	14	16	14	12	20	9	91	100	2	0.98	2	512
11	12	14	26	19	19	17	18	34	14	129	100	1	0.99	2	462
12	13	10	23	16	19	18	14	32	14	122	100	2	1.11	2	511
14	13	10	23	17	20	16	13	31	17	122	100	2	0.96	2	444
15	14	12	26	18	19	15	17	30	19	126	90	2	0.85	1	297
16	20	15	35	12	14	13	11	20	16	91	90	2	1.07	2	436
17	15	14	29	11	14	14	11	25	14	94	100	2	1.14	2	473
18	15	12	27	11	19	16	14	28	16	111	60	1	0.85	1	510
19	17	14	31	14	14	14	12	24	12	96	90	1	1.01	2	351
20	14	10	24	19	17	16	15	30	19	124	97	1	1.17	2	307

**Table 5:** Outcomes at the 6-month follow-up (T2).

ID	Anxiety	Depression	Negative emotion	Physical domain	Psychological domain	Independence domain	Social domain	Environment domain	Spirituality domain	Quality of life	VAS	Self-report adherence	MPR	MPR (binary)	CD4 (/µL)
1	15	14	29	18	19	18	14	34	17	128	100	2	0.93	1	532
2	15	15	30	14	15	12	11	25	12	94	100	2	1.29	2	498
3	16	20	36	10	11	12	11	22	14	85	100	2	0.99	2	479
4	7	7	14	19	24	18	17	37	20	145	90	2	0.99	2	488
5	9	7	16	16	20	15	15	29	19	120	90	2	1.1	2	506
6	15	14	29	15	16	14	14	26	16	107	90	2	0.99	2	396
7	20	10	30	17	19	20	13	33	11	120	99	1	1.43	2	908
8	15	16	31	11	15	12	11	25	15	95	100	2	1.96	2	430
9	7	9	16	17	21	19	16	35	19	135	90	2	1.02	2	501
10	12	8	20	13	15	15	12	20	17	98	90	2	1.06	2	512
11	11	12	23	17	21	16	16	32	19	129	100	2	1.07	2	455
14	12	11	23	16	21	17	15	31	17	125	100	2	1.45	2	585
15	14	10	24	18	21	16	15	30	17	125	100	2	1.18	2	286
16	18	18	36	13	12	13	10	19	15	87	90	2	0.94	1	393
17	14	11	25	12	15	11	11	23	15	93	80	2	1.3	2	481
18	14	14	28	14	19	16	13	30	19	119	50	1	0.93	1	541
19	15	12	27	14	15	14	12	27	14	102	95	2	0.99	2	293
20	14	11	25	20	17	15	15	31	16	122	100	1	0.92	1	359

**Table 6:** Exposure variables (Attendance rates).

ID	Sessions										Attendance
	1	2	3	4	5	6	7	8	9	10	
1	√	√	√	√	√	√	√	√	√	√	10
2	√	√	√	√	√	√	√	√	√	√	9
3	√		√		√	√	√		√	√	7
4		√		√	√	√	√	√	√		7
5	√	√	√			√	√		√		6
6	√	√	√	√		√	√	√	√	√	9
7		√									1
8	√										1
9			√	√	√		√				4
10	√	√	√	√		√		√			6

Among the 20 participants, all were males, with a mean age of 36.15 years old. Most of the participants (60%) did not have a Shanghai household registry, which means that they were migrants from other areas. The majority of the participants (95%) were of Han ethnicity. Most of them had a high educational level, with 80% of them having a bachelor's degree or higher. Approximately half of the participants had a religious affiliation. Most of them were employed (90%). Only 4 participants were married (20%) (Table 1).

Of the total sample, the participants showed an average PHQ-4 score of 5.00, an average year of diagnosis of 4.20 years, and average years of receiving ART of 3.80 years. Half of the participants (50%) had CD4 lymphocyte counts of more than 400/µL, with an average count of 405/µL. All had the latest undetectable virus load (100%). Most of them (80%) were infected with HIV because of homosexual behavior (Table 2).

Among all the outcomes, all are continuous variables except the self-report adherence and the MPR. After a 10-week intervention, one participant in the control group refused to continue follow-up (Table 4). Another participant in the control group was unable to be contacted after the 6-month follow-up (Table 5). No one dropped out of the intervention group. The sample retention rate was 90%.

According to our sign-in record, within the intervention group, one participant attended ten sessions; two participants attended nine sessions; two participants attended seven sessions; two participants attended six sessions; one participant attended four sessions; two participants attended one session. The overall attendance rate was 60% (Table 6).

## 2 Experimental design, materials, and methods

A randomized controlled trial was conducted to examine the preliminary effects of group CBI on depression (primary outcome), anxiety, quality of life, medication adherence, and CD4 lymphocyte count (secondary outcomes). This study was approved by the Research Ethical Committee of the School of Nursing, Fudan University (IRB#TYSA2016-3–1), and Shanghai Public Health Center Affiliated with Fudan University (2019-S036-02). It was also registered with the Chinese Clinical Trial Registry (ChiCTR1900024256).

Participants were eligible if they met the following inclusion criteria: ①Participants who had been diagnosed with HIV-1 infection; ②more than 18 years old; ③receiving anti-retroviral treatment (ART); ④the Patient Health Questionnaire-4 (PHQ-4) score≥2. Participants were excluded if they ①could not participate in our study because of severe comorbidities or cognitive impairment and ②were taking part in other HIV-related research projects at the same time.

Participants were assigned in the control or the intervention group randomly. We used the WPS EXCEL software to generate the random sequence and sorted the random number by size. After the eligible participants signed the informed consent form, they were asked to open an envelope that contained the information about the assigned group. The data collection staff was blinded.

We designed the “1 + 2 + 10” strategy in the intervention group, which means one nurse, two voluntary assistant intervention providers with psychological background, and ten participants in the CBT group. Table 7 summarizes the content of the intervention sessions.

**Table 7:** Content of intervention sessions.

Session	Didactic components	Activity/Topic
1	An explanation for group goals, physiological effects of stress	Ice-breaking activity, collection of questions and expectations
2	Conception and interpretation of CBT	What dose HIV infection mean to me?
3	Definition and practice skills of mindfulness	Why am I a gay?
4	Identification of cognitive distortions and automatic thoughts	Intimate relationships
5	Rational thought replacement	How to minimize the impact of medication on life?
6	Coping skills training	What will I do when physicians reject me?
7	Assertiveness training	Career development
8	Emotion management	Fake marriage and surrogacy
9	Identification of social support	HIV confidentiality and notification
10	Summary	Look into the mirror

## Appendix Supplementary materials

Image, application 1

Supplementary questionnaire Image, application 2
