# Supplementary material for: Dataset of cognitive behavioral intervention for persons living with HIV in China: A randomized pilot trial
Source: Data Brief. 2020 Mar 31;30:105459. doi: 10.1016/j.dib.2020.105459 (PMC7153287; doi:10.1016/j.dib.2020.105459)
Supplement: Supplementary file 1 [file mmc1.doc]

**PHQ-4**

Over the last 2 weeks, how often have you been bothered by the following problems? (use “√” to indicate your answer)

|  | Not at all | Several days | More than half the days | Nearly every day |
| --- | --- | --- | --- | --- |
| **Feeling nervous, anxious or on edge** | 0 | 1 | 2 | 3 |
| **Not being able to stop or control worrying** | 0 | 1 | 2 | 3 |
| **Little interest or pleasure in doing things** | 0 | 1 | 2 | 3 |
| **Feeling down, depressed, or hopeless** | 0 | 1 | 2 | 3 |

**Sociodemographic information**  Treatment ID：_________

1. **Date of birth:** ___________Year___________Month

**2. Gender：**□Male □Female

**3. Race：**□Han □Minority

**4. Education level:**

□Primary school or less □Junior middle school □Senior high school

□College □University □Master’s or above

1. **Religion:** □Yes □No

**6. Employment status:** □Yes □No

**7. Marital status:** □Single □Married

**8. Transmission mode：**□Homosexual behavior □Heterosexual behavior □other

**9.** **The latest CD4+ T-cell count:** ________________

**10. The latest virus load:** ________________

**11. Date of HIV diagnosis：**___________Year___________Month

**12. Date of starting antiretroviral therapy：**___________Year___________Month

**13. Household registry：**□Shanghai □Other

**Medication adherence tests**

1. In the past month, did you miss taking medication or mistakenly take medication?

□Yes（number of times： ）□No

2. In the past month, did you delay or take medication ahead of time by more than 2 hours?

□Yes（number of times： ）□No

3. If a score of 0 means no medication at all, and a score of 100 means taking all the medication as required by your doctor, how much do you rate your medication over the past month?


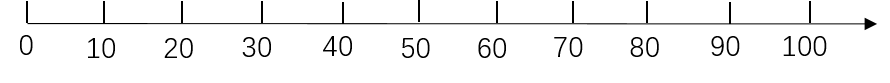


**Hospital Anxiety and Depression Scale (HADS)**

***Instructions***

Tick the box beside the reply that is closest to how you have been feeling in the past week. Don’t take too long over you replies: your immediate is best.

**1. I feel tense or ‘wound up’:**

□Not at all □From time to time □A lot of the time □Most of the time

**2. I still enjoy the things I used to enjoy:**

□Definitely as much □Not quite so much □Only a little □Hardly at all

**3. I get a sort of frightened feeling as if something awful is about to happen:**

□Not at all □A little, but it doesn’t worry me

□Yes, but not too badly □Very definitely and quite badly

**4. I can laugh and see the funny side of things:**

□As much as I always could □Not quite so much now

□Definitely not so much now □Not at all

**5. Worrying thoughts go through my mind:**

□Only occasionally □From time to time, but not too often □A lot of the time □A great deal of the time

**6. I feel cheerful**

□Most of the time □Sometimes □Not often □Not at all

**7. I can sit at ease and feel relaxed:**

□Definitely □Usually □Not often □Not at all

**8. I have lost interest in my appearance:**

□I take just as much care as ever □I may not take quite as much care

□I don’t take as much care as I should □Definitely

**9. I feel restless as I have to be on the move:**

□Not at all □Not very much □Quite a lot □Very much indeed

**10. I look forward with enjoyment to things:**

□As much as I ever did □Rather less than I used to

□Definitely less than I used to □Hardly at all

**11. I get sudden feelings of panic:**

□Not at all □Not very often □Quite often □Very often indeed

**12. I feel as if I am slowed down:**

□Not at all □Sometimes □Very often □Nearly all the time

**13. I get a sort of frightened feeling like ‘butterflies’ in the stomach:**

□Not at all □Occasionally □Quite often □Very often

**14. I can enjoy a good book or radio or TV program:**

□Often □Sometimes □Not often □Very seldom

Note: Anxiety score = Q1 + Q3 + Q5 + Q7 + Q9 + Q11 + Q13, range from 7 to 28;

Depression score = Q2 + Q4 + Q6 + Q8 + Q10 + Q12 + Q14, range from 7 to 28.

The higher the score, the worse the situation is.

**WHOQOL-HIV BREF**

***Instructions***

This assessment asks how you feel about your quality of life, health, or other areas of your life. If you are unsure about which response to give to a question, **please choose the one** that appears most appropriate. This can often be your first response. Please keep in mind your standards, hopes, pleasures and concerns. We ask that you think about your life **in the last two weeks.**

1. **How would you rate your quality of life？**

□Very poor □Poor □Neither poor nor good □Good □Very good

**2. How satisfied are you with your health？**

□Very dissatisfied □Dissatisfied □Neither satisfied nor dissatisfied

□Satisfied □Very satisfied

**3. How well are you able to get around？**

□Very poor □Poor □Neither poor nor good □Good □Very good

**4. How often do you have negative feelings such as blue mood, despair, anxiety, depression in the last two weeks?**

□Never □Seldom □Quite often □Very often □Always

|  | Not at all | A little | A moderate amount | Very much | An extreme amount |
| --- | --- | --- | --- | --- | --- |
| 5.To what extent do you feel that physical pain prevents you from doing what you need to do？ |  |  |  |  |  |
| 6.How much are you bothered by any physical problems related to your HIV infection？ |  |  |  |  |  |
| 7.How much do you need any medical treatment to function in your daily life？ |  |  |  |  |  |
| 8.How much do you enjoy life？ |  |  |  |  |  |
| 9.To what extent do you feel your life to be meaningful？ |  |  |  |  |  |
| 10.To what extent are you bothered by people blaming you for your HIV status？ |  |  |  |  |  |
| 11.How much do you fear the future？ |  |  |  |  |  |
| 12.How much do you worry about death？ |  |  |  |  |  |
|  | Not at all | A little | A moderate amount | Very much | Extremely |
| 13.How well are you able to concentrate？ |  |  |  |  |  |
| 14.How safe do you feel in your daily life？ |  |  |  |  |  |
| 15.How healthy is your physical environment？ |  |  |  |  |  |
|  | Not at all | A little | Moderately | Mostly | Completely |
| 16.Do you have enough energy for everyday life？ |  |  |  |  |  |
| 17.Are you able to accept your bodily appearance？ |  |  |  |  |  |
| 18.Have you enough money to meet your needs？ |  |  |  |  |  |
| 19.To what extent do you feel accepted by the people you know？ |  |  |  |  |  |
| 20.How available to you is the information that you need in your day-to-day life？ |  |  |  |  |  |
| 21.To what extent do you have the opportunity for leisure activities？ |  |  |  |  |  |
|  | Very dissatisfied | Dissatisfied | Neither satisfied nor dissatisfied | Satisfied | Very satisfied |
| 22.How satisfied are you with your sleep？ |  |  |  |  |  |
| 23.How satisfied are you with your ability to perform your daily living activities？ |  |  |  |  |  |
| 24.How satisfied are you with your capacity for work？ |  |  |  |  |  |
| 25.How satisfied are you with yourself？ |  |  |  |  |  |
| 26.How satisfied are you with your personal relationship？ |  |  |  |  |  |
| 27.How satisfied are you with your sex life？ |  |  |  |  |  |
| 28.How satisfied are you with the support you get from your friends？ |  |  |  |  |  |
| 29.How satisfied are you with the conditions of your living place？ |  |  |  |  |  |
| 30.How satisfied are you with your access to health services？ |  |  |  |  |  |
| 31.How satisfied are you with your transport？ |  |  |  |  |  |

Note: Physical domain = (6 - Q5) + (6 - Q6) + Q16 + Q22, range from 4 to 20;

Psychological domain = (6 - Q4) + Q8 + Q13 + Q17 + Q25, range 5 to 25;

Independence domain = Q3 + (6 - Q7) + Q23 + Q24, range from 4 to 20;

Social domain = Q19 + Q26 + Q27 + Q28, range from 4 to 20;

Environment domain = Q14 + Q15 + Q18 + Q20 + Q21 + Q29 + Q30 + Q31, range from 8 to 40;

Spirituality domain = Q9 + (6 - Q10) + (6 - Q11) + (6 - Q12), range from 4 to 20;

Quality of life = Physical domain + Psychological domain + Independence domain + Social domain + Environment domain + Spirituality domain + Q1 + Q2, range from 31 to 155.

The higher the score, the better the situation is.
